# The role of cerebellar circuitry alterations in the pathophysiology of autism spectrum disorders

**DOI:** 10.3389/fnins.2015.00296

**Published:** 2015-09-01

**Authors:** Matthew W. Mosconi, Zheng Wang, Lauren M. Schmitt, Peter Tsai, John A. Sweeney

**Affiliations:** ^1^Clinical Child Psychology Program and Schiefelbusch Institute for Life Span Studies, University of KansasLawrence, KS, USA; ^2^Center for Autism and Developmental Disabilities, University of Texas SouthwesternDallas, TX, USA; ^3^Department of Psychiatry, University of Texas SouthwesternDallas, TX, USA; ^4^Department of Pediatrics, University of Texas SouthwesternDallas, TX, USA; ^5^Department of Neurology and Neurotherapeutics, University of Texas SouthwesternDallas, TX, USA; ^6^Department of Neuroscience, University of Texas SouthwesternDallas, TX, USA

**Keywords:** autism spectrum disorder, cerebellum, sensorimotor, genetics, pathophysiology, oculomotor, precision grip, gait

## Abstract

The cerebellum has been repeatedly implicated in gene expression, rodent model and post-mortem studies of autism spectrum disorder (ASD). How cellular and molecular anomalies of the cerebellum relate to clinical manifestations of ASD remains unclear. Separate circuits of the cerebellum control different sensorimotor behaviors, such as maintaining balance, walking, making eye movements, reaching, and grasping. Each of these behaviors has been found to be impaired in ASD, suggesting that multiple distinct circuits of the cerebellum may be involved in the pathogenesis of patients' sensorimotor impairments. We will review evidence that the development of these circuits is disrupted in individuals with ASD and that their study may help elucidate the pathophysiology of sensorimotor deficits and core symptoms of the disorder. Preclinical studies of monogenetic conditions associated with ASD also have identified selective defects of the cerebellum and documented behavioral rescues when the cerebellum is targeted. Based on these findings, we propose that cerebellar circuits may prove to be promising targets for therapeutic development aimed at rescuing sensorimotor and other clinical symptoms of different forms of ASD.

## Cerebellar pathology in autism spectrum disorder

The majority of *in vivo* brain studies of individuals with autism spectrum disorder (ASD) have focused on neural networks involved in social behavior, language, and behavioral and cognitive flexibility—the defining features of the disorder (American Psychiatric Association, 2013). Still, the full extent of neural systems impacted by ASD is not yet well understood, and pathophysiological mechanisms associated with the disorder remain elusive. There are multiple factors that have limited progress toward identifying brain mechanisms in ASD including the complexity of the psychological/behavioral constructs that have been most systematically investigated (e.g., theory of mind processing), limited knowledge about their neural underpinnings, clinical, and neurobiological heterogeneity across the autism spectrum, a lack of integration of knowledge about the developmental neurobiology of relevant brain systems, and failures to link *in vivo* case-control studies of psychological dimensions with what is known about histopathological and molecular mechanisms associated with ASD.

The cerebellum remains an understudied area in clinical investigations of ASD. It is perhaps the most consistently implicated brain region in post-mortem studies. Reports have indicated 35–95% fewer cerebellar Purkinje cells in ASD brains compared to controls (Bauman and Kemper, 1985; Arin et al., 1991; Bailey et al., 1998; Whitney et al., 2008, 2009; Wegiel et al., 2014), and remaining cells appear to be reduced in size (Fatemi et al., 2002a). The majority of cases studied to date (30/45) show reduced Purkinje cell density in posterior lateral hemispheres of the cerebellum, but fewer studies have found these anomalies in the vermis. A recent examination of eight patients found that Purkinje cell density reductions were more severe in Crus I–II, but that they were still present in lobules IV–VI and lobule X as well (Skefos et al., 2014). Deep cerebellar nuclear cells to which Purkinje cells project also are abnormal in ASD showing enlargement during childhood and subsequent reductions in size and number during adolescence and adulthood (Bauman, 1991). Therefore, patterns of cerebellar pathology may be regionally specific as well as variable across development. Levels of glutamic acid decarboxylase (GAD) 65 and 67 proteins involved in converting glutamate to GABA are reduced in the cerebella of individuals with ASD (Fatemi et al., 2002b; Yip et al., 2007, 2008). Reductions in GABA_A_α1 protein levels and GABA_B_ R1 receptor density in cerebella of ASD patients also have been documented (Fatemi et al., 2010).

Studies of etiopathologic mechanisms associated with ASD have consistently implicated the cerebellum as well. Computational studies have found that ASD susceptibility genes are co-expressed in human cerebellum between the neonatal period and age 6 years (Willsey et al., 2013), particularly within the granule cell layer (Menashe et al., 2013). Further, many syndromic forms of ASD involve cerebellar alterations including Phelan-McDermid Syndrome, Fragile X Syndrome (FXS), Tuberous Sclerosis (TSC), and patients with 15q11 duplication syndrome (Abrahams and Geschwind, 2010; Mosconi et al., 2011; Kloth et al., 2015). Cerebellar alterations also appear to be specifically associated with ASD features. For example, while FXS is associated with ASD and involves disruptions of multiple brain systems, posterior vermis lobules VI–VII are affected only in individuals with FXS with comorbid ASD (Kaufmann et al., 2003). In the context of structural MRI studies showing that posterior vermis lobules VI–VII also are reduced in volume in idiopathic ASD, these findings provide strong evidence that posterior vermal alterations may be uniquely associated with ASD (see Stanfield et al., 2008 for a meta-analysis). Additional evidence for cerebellar anomalies being selectively involved in ASD comes from studies of individuals with TSC, a genetic disorder caused by mutations of either TSC1 or TSC2 genes and characterized by hamartomas in the brain and other organs. Approximately 40% of individuals with TSC also are diagnosed with ASD, and those individuals with TSC and cerebellar lesions have more severe ASD features than those with lesions affecting other brain regions (Eluvathingal et al., 2006). Studies of children who have experienced perinatal cerebellar injuries further support a central role of the cerebellum in the development of ASD. These children experience a 36-fold increased risk of developing ASD, making perinatal cerebellar damage the greatest known non-genetic risk factor associated with the disorder (Bolduc and Limperopoulos, 2009; Limperopoulos et al., 2009; Bolduc et al., 2011; Wang et al., 2014).

Despite evidence for a primary role of the cerebellum in the pathophysiology of ASD, the literatures describing the cerebellar circuitries that are affected in patients and how they relate to clinical impairments remain in their infancy. Afferent processes to pontine nuclei originate from widespread regions of neocortex and are relayed to different lobules of cerebellar cortex via mossy fiber inputs (Eccles et al., 1967). These inputs arrive from motor, sensory, posterior parietal, prefrontal, cingulate, orbitofrontal, and temporal cortices as well as basal ganglia nuclei (Dum and Strick, 2003). Output from the deep nuclei of the cerebellum (dentate, interpositus, and fastigial) innervate different subdivisions of ventrolateral thalamus (Percheron et al., 1996) and then project to multiple neocortical areas (Leiner et al., 1991, 1993). These cortical-pontine-cerebellar-thalamic-cortical loops are highly segregated and support distinct behavioral and cognitive functions including sensorimotor, language, affective, and executive abilities (Habas et al., 2009; Krienen and Buckner, 2009). Defects of the cerebellum thus could have a pervasive impact on behavioral and cognitive development while increasing risk for ASD by disrupting the maturation and function of these cortical-cerebellar loops. If pathology in the cerebellum is localized, cerebellar anomalies could have a selective impact on different circuitries and thus contribute to symptom heterogeneity in ASD.

There is accumulating evidence that multiple cortical-cerebellar circuits are anatomically and functionally abnormal in patients with ASD (Table 1). Diffusion tensor imaging (DTI) studies have identified white matter alterations of the primary output pathway from the cerebellum, the superior cerebellar peduncle, and the primary cortical input pathway to the cerebellum, the middle cerebellar peduncle (Catani et al., 2008; Shukla et al., 2010; Sivaswamy et al., 2010). While these studies suggest that cerebellar input and output processes connecting it to neocortical areas are atypical in ASD, current DTI methods are not able to discern the extent to which these anomalies selectively involve different cortical-cerebellar loops.

**Table 1:** **Summary of findings from prior ASD studies of different sensorimotor cerebellar circuits**.

	**Oculomotor circuits**	**Upper limb circuits**	**Gait/Posture circuits**
Histopathology	Minority of cases show reduced PC density in vermis, though not as prominent as in hemispheres	Consistent reports of reduced PC density in lob. IV–VI extending into lateral areas including Crus I–II	Reduced PC density in spinocerebellum, though less prominent than in anterior or lateral lobs
MRI anatomy	Findings of vermal hypoplasia involving lobules VI–VII	Consistent findings of overgrowth throughout hemispheres	Reduced volumes found in inferior lob. IX
Sensorimotor behavior	Increased amplitude of saccadic intrusions during gaze fixation Reduced saccade accuracy Increased saccade amplitude variability Reduced rates of saccade adaptation Reduced closed-loop smooth pursuit gain Reduced gain of rightward eye movements during open-loop phase of smooth pursuit (first 100 ms)	Atypical reaching kinematics Slower, less smooth reach-to-grasp movements Reduced coordination of grip and lift forces during grasping Excess grip force during initial gripping Increased force variability during sustained gripping Increased reliance on proprioceptive feedback during motor learning processes Impairments in visual feedback processing during gripping and motor learning	Reduced anterior postural adjustments during self-timed loading/unloading Increased postural sway, esp. in mediolateral directions, during quiet stance Reduced postural sway when trying to initiate and maintain a dynamic stance Increased stride width, decreased stride length, atypical walking kinematics
Functional imaging	Reduced BOLD activation during saccades and smooth pursuit eye movements	Reduced and increased anterior and lateral cerebellar BOLD activity found during finger tapping and button pressing sequencing tasks	No studies reported

*PC, Purkinje cells; ms, milliseconds; lob, lobule(s)*.

A recent meta-analysis highlights a unique profile of volumetric reductions in cerebellar gray matter of individuals with ASD that is distinct from alterations found in attention deficit-hyperactivity disorder (ADHD) and developmental dyslexia (Stoodley, 2014). ASD-specific reductions in volume were found in inferior lobule IX, left lobule VIIIB, and Crus I. These regions also showed functional connectivity with frontoparietal, default mode, somatomotor, and limbic areas, consistent with the idea that different forms of cerebellar pathology may differentially impact multiple brain networks and cause varying developmental dysfunctions. Accordingly, atypical patterns of cortical-cerebellar activation and functional connectivity have been demonstrated in ASD during tests of simple motor skills (Mostofsky et al., 2009), language (Hodge et al., 2010; Verly et al., 2014), and emotion processing (Critchley et al., 2000). Importantly, a recent resting state fMRI study of individuals with ASD documented overconnectivity of cortical-cerebellar circuits involved in sensorimotor control, as well as underconnectivity of cerebellar circuits involved in cognitive and higher-order operations (Khan et al., 2015). These findings provide important functional evidence that while cerebellar pathology in ASD may affect multiple cortical and deep nuclear circuits, distinct cortical-cerebellar loops or circuits may be altered in different ways.

Studies of sensorimotor behaviors offer perhaps the most direct approach for understanding the functional integrity of different cerebellar circuits in ASD patients. Sensorimotor tasks are highly translational, precisely quantifiable in both spatial and temporal domains, and readily studied across wide age ranges and developmental levels. The cerebellar networks supporting sensorimotor development are relatively well defined and include motor and parietal cortices as well as basal ganglia nuclei (Gazzaniga and Mangun, 2014)—regions that have been repeatedly implicated in ASD (Sears et al., 1999; Stanfield et al., 2008; Mostofsky et al., 2009; Wolff et al., 2013).

Sensorimotor control is understudied in ASD, but there is a growing literature showing that cerebellar-dependent sensorimotor behaviors are compromised in ASD patients. Sensorimotor deficits are found in the majority of individuals with ASD (Fournier et al., 2010a), and these impairments can emerge and be detected as early as infancy (Brian et al., 2008; Lebarton and Iverson, 2013; Ben-Sasson and Gill, 2014; Leonard et al., 2014; Ozonoff et al., 2014; Sacrey et al., 2015). They also appear to be familial, suggesting that they may serve as useful endophenotypes for advancing gene discovery (Mosconi et al., 2010). In the present paper, we will describe what is known about the distinct cerebellar circuitries involved in sensorimotor behaviors and their functional integrity in ASD. Approaches for understanding these circuitries in animal models of ASD and determining their utility as targets for treatment development also will be discussed.

## Cerebellar circuits supporting sensorimotor behaviors

More than half of all mature neurons in the brain are located in the cerebellum (Butts et al., 2012), and many of the circuits formed by these cells are involved in various aspects of motor control (Ito, 1984). The precise role of these circuits in controlling motor behavior remains debated, as various zones of the cerebellum appear to make distinct contributions including controlling the timing of movements, simultaneously coordinating the movement dynamics of different effectors (e.g., shoulder, elbow, and wrist joints during reaching), and integrating multiple cortical signals and sensory inputs (Holmes, 1917; Bower, 2002; Jacobson et al., 2008; D'angelo and De Zeeuw, 2009; De Zeeuw et al., 2011). One prominent and unifying framework hypothesizes that the cerebellum serves as a forward controller of motor and cognitive activity (Ito, 1983, 2008; Miall and Reckess, 2002). According to this model, the cerebellum provides forward models used to predict the position and motion of body parts based on an internal model of the dynamics required to complete a given task. Forward control allows for rapid action as sensory feedback processes often occur too slowly to guide initial or dynamic movements. After a motor command is generated by the motor cortex, a copy of this command is sent to the cerebellum (efference copy). The cerebellum then uses its forward model to predict the sensory consequences of the action (corollary discharge). The sensory predictions are subsequently compared to actual sensory feedback and the cerebellum generates corrective commands to refine the ongoing movement (Wolpert et al., 1998). When the action repeatedly deviates from the expected outcome, the forward model of the cerebellum undergoes refinement to ensure the accuracy of subsequent output (Scudder, 2002; Izawa et al., 2012a; Herzfeld et al., 2014).

**Figure 1:**
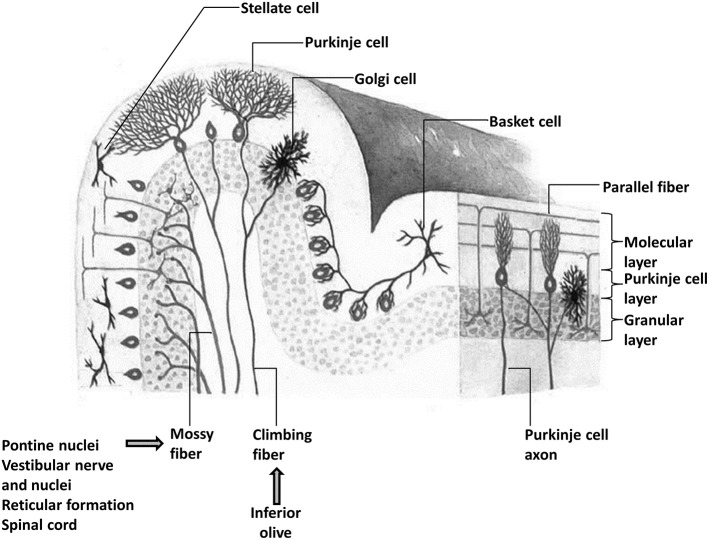
**The microstructural organization of the cerebellar cortex showing the presence of three layers and the relative position of Purkinje, basket, stellate, and Golgi cells and the main inputs (mossy and climbing fibers) of the cerebellum**. There are two main afferents to the cerebellar cortex: climbing fibers, which make direct excitatory contact with the Purkinje cells, and mossy fibers, which terminate in the granular layer and make excitatory synaptic contacts primarily with granule cells, but also with Golgi cells. The ascending axons of the granule cells branch in a T-shaped manner to form the parallel fibers, which, in turn, make excitatory synaptic contacts with Purkinje cells and molecular layer interneurons including stellate and basket cells.

A unifying framework for the cerebellum appears plausible in the context of its relatively invariant cellular architecture that contrasts the diversity of cellular composition across different neocortical areas. The cerebellar cortex consists of numerous “microcomplexes” that are structured similarly across lobules and different subregions (Ito, 2008) (Figure 1). These microcomplexes are comprised of mossy fiber inputs, primarily originating from pontine nuclei but also from vestibular nuclei, the spinal cord, and reticular formation. Mossy fibers innervate Purkinje cells of the cerebellar cortex via granular cells and parallel fibers as well as feedback collaterals from deep cerebellar nuclei. Excitatory mossy fiber inputs provide information from neocortical regions as well as the spinal cord (Vogel et al., 1996; Ramnani, 2006; Geborek et al., 2014). Climbing fibers originating in the inferior olive communicate instructional or “teaching” signals directly to Purkinje cells and initiate a process of long-term depression (LTD) that selectively prunes parallel fiber-Purkinje cell synapses and modifies the strength of inhibitory output from Purkinje cells to deep nuclei (Nguyen-Vu et al., 2013). This process is the basis of cerebellar learning and it allows the cerebellum to consolidate and then modify internal models of action that are used to predictively control motor behavior (Wolpert et al., 1998).

**Figure 2:**
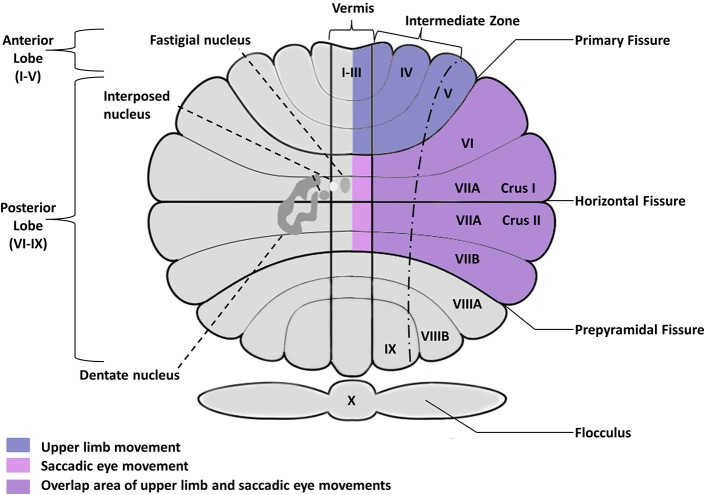
**Posterior view of the human cerebellum, showing the cerebellar fissure, lobular organization, and deep nuclei embedded within the cerebellar cortex**. Deep nuclei are located bilaterally but shown only in the left hemisphere for clarity purposes. Saccadic and smooth pursuit eye movements are controlled by the oculomotor vermis including posterior lobules VI–VII, Crus I–II of the ansiform lobule, and their outputs in caudal fastigial nuclei. Upper limb movements primarily involve anterior lobules I–V as well as more lateral areas of lobules V–VI extending into Crus I–II. Cerebellar circuits involved in controlling balance and gait have been identified in the vermis and intermediate cerebellum (not shown).

Despite the similarity of these learning units across the cerebellar hemispheres and vermis, there is considerable anatomical specificity for different types of movements and different aspects of movement control (Figure 2). Within the cerebellum, somatic representations similar to those localized in motor cortex have been demonstrated both in the cerebellar cortex and in the deep nuclei (see Manni and Petrosini, 2004 for a review). Microzones consisting of parasagittally aligned Purkinje cell populations form functional units that innervate discrete areas of deep cerebellar nuclei and receive segregated projections from the inferior olive (Cerminara, 2010; Oberdick and Sillitoe, 2011). Thus, the geometry of the cerebellum is largely invariant at the cellular level, but highly specialized and segregated functional units are found at an intermediate level and at the level of cortical-cerebellar networks.

The distinct cerebellar regions that support different types of motor behavior have been well described in human imaging and lesion studies as well as single-cell recordings of non-human primates. These studies have identified discrete circuits supporting eye movements, limb movements, and posture/gait. Saccadic eye movements, or rapid shifts in eye gaze, as well as smooth pursuit eye movements are controlled by the oculomotor vermis including posterior lobules VI–VII, Crus I–II of the ansiform lobule, and their outputs in caudal fastigial nuclei (Takagi et al., 1998; Alahyane et al., 2008; Panouillères et al., 2012). Crus I–II of ansiform lobule, the flocculus and paraflocculus, uvula and nodulus are critically involved in steady gaze fixation, smooth pursuit eye movements, and the vestibular-ocular reflex (VOR) that is used to maintain fixation during head rotation (Robinson et al., 1993; Hashimoto and Ohtsuka, 1995; Baier et al., 2009). Upper limb movements are under the supervision of the intermediate and lateral zones of the cerebellar cortex and their targets in the interposed and dentate nuclei (Thach et al., 1992; Thach, 1997; Kuper et al., 2012; Maderwald et al., 2012; Stefanescu et al., 2013). Circuits located more medial in the vermis and intermediate cerebellum receive neocortical input as well as direct spinal input to control balance and gait (Brooks and Thach, 1981; Sullivan et al., 2010; Vassar and Rose, 2014). Further, there appear to be distinct subregions within these circuits to control different aspects of motor output. For example, Neely et al. (2013) found that cerebellar areas controlling initial manual motor output based on internal action representations appear to be anterior to those involved in continuous control of motor behavior based on visual feedback. The high degree of functional specialization of these distinct circuits suggests that their study in ASD may provide key insights into the developmental neurobiology of this disorder and the pathogenesis of sensorimotor issues and perhaps broader behavioral and cognitive deficits.

## Oculomotor control in ASD

Studies of oculomotor control may be highly informative regarding cerebellar function in ASD owing to their well-defined neurophysiological substrates, quantitative nature, high degree of heritability (Bell et al., 1994), and stability over time (Yee et al., 1998; Reilly et al., 2005; Irving et al., 2006; Lencer et al., 2008). Abnormalities of eye gaze also are part of the diagnostic criteria for ASD, and while these deficits have been well studied during social interactions, it is possible that more fundamental and earlier emerging alterations of oculomotor control could contribute to atypical patterns of eye gaze coordination among affected children (Bryson et al., 2007; Elison et al., 2013).

Gaze fixation is an active process used to stabilize the fovea on an image or object. While the eye undergoes naturally occurring drift during the process of visual fixation, the oculomotor system generates microsaccades to counter this drift and maintain fixation (Zuber et al., 1965; Epelboim and Kowler, 1993). Visual fixation is supported by the reciprocal balance of excitatory burst and inhibitory omnipause neurons within the pons of the brainstem as well as inputs from the frontal eye fields and superior colliculus that actively suppress saccades away from the object of interest (Leigh and Zee, 2006). Pontine nuclei innervate Purkinje cells of cerebellar vermis lobules VI–VII, and inhibitory output from the oculomotor vermis helps suppress unwanted eye movements and maintain an image on the fovea (Kase et al., 1980). Abnormalities during visual fixation including slow, large amplitude ocular drift, square wave saccadic intrusions, and gaze-evoked nystagmus (repetitive, to-and-fro movements of the eyes) each have been documented in patients with cerebellar lesions (Jeong et al., 2007; Serra et al., 2008; Shaikh et al., 2009; Baier and Dieterich, 2011).

Structural MRI and post-mortem studies have documented abnormalities of the pons (Gaffney et al., 1988; Hashimoto et al., 1991, 1993; Hashimoto and Ohtsuka, 1995; Bailey et al., 1998; Jou et al., 2013) and cerebellar vermal lobules VI–VII in ASD (Courchesne et al., 1988; Murakami et al., 1989; Hashimoto et al., 1995; Fatemi et al., 2002a; Stanfield et al., 2008). Studies of visual fixation in ASD have demonstrated increased amplitude and reduced inter-saccade intervals of square-wave jerk saccades relative to healthy controls (Nowinski et al., 2005; Aitkin et al., 2013) suggesting increased excitation in ponto-cerebellar circuitry in patients.

Saccades, or rapid shifts in eye gaze, are controlled by highly specialized cortical-cerebellar circuits that also involve posterior vermis and caudal regions of fastigial nuclei. The initiation of saccades relies on the inverse process of visual fixation control via the pons described above. In order for a saccade to be initiated, the tonic inhibition of pontine burst cells must be simultaneously released by omnipause cells while also being driven by excitatory signals from the superior colliculus (Robinson, 1975; Leigh and Zee, 2006). The dynamics of saccadic eye movements are directly related to the firing rates of the burst cells and their interactions with cerebellar output that predictively controls the amplitude and accuracy of the movement (Luschei and Fuchs, 1972; Van Gisbergen et al., 1981; Yoshida et al., 1999).

Reduced accuracy (Rosenhall et al., 1988; Takarae et al., 2004b; Luna et al., 2007; Johnson et al., 2012; Schmitt et al., 2014) and increased trial-to-trial accuracy variability (Takarae et al., 2004b; Stanley-Cary et al., 2011; Johnson et al., 2012; Schmitt et al., 2014) of saccadic eye movements have been repeatedly documented in individuals with ASD. These results implicate forward control mechanisms of the oculomotor vermis in ASD and a reduced ability to precisely update internal representations used for forward control. This profile of deficits in saccade control is similar to what is seen in non-human primates following ablation of the oculomotor vermis (Takagi et al., 1998) and patients with spinocerebellar (Federighi et al., 2011) and Friedreich's Ataxia (Kirkham et al., 1979). Studies of saccade dynamics in ASD have identified reduced velocities (Johnson et al., 2012; Schmitt et al., 2014), increased duration (Rosenhall et al., 1988; Stanley-Cary et al., 2011; Schmitt et al., 2014), and prolonged periods of movement acceleration during the saccade (Schmitt et al., 2014) suggesting an imbalance of pontine excitatory and inhibitory processes that reciprocally interact with the cerebellum.

In the only known ASD study to directly examine brain systems underlying saccade control, Takarae et al. (2007) used fMRI to show reduced activation of frontal eye fields, posterior parietal cortex, and cerebellar vermis and hemisphere lobules VI–VII in patients making visually guided saccades. Individuals with ASD also demonstrated increased activation within frontal-striatal regions including the thalamus, caudate nucleus, dorsolateral prefrontal cortex, and anterior cingulate cortex. These results provide direct evidence for cortical-cerebellar dysfunctions during eye movements in ASD, and also suggest that frontostriatal systems typically dedicated to higher-order cognitive processes may become more involved in simple motor actions to compensate for cortical-cerebellar alterations in ASD patients.

The oculomotor vermis also is involved in controlling smooth pursuit eye movements used to track slowly moving targets. Smooth visual pursuit relies on the rapid and temporally precise integration of information from multiple brain regions including extrastriate areas of the visual cortex responsible for processing visual motion, cortical eye fields and the cerebellum responsible for translating sensory information into motor commands, and the striatum and brainstem responsible for initiating motor commands (Lisberger et al., 1987; Keller and Heinen, 1991; Ilg, 1997; Berman et al., 1999; Rosano et al., 2002). The initial phase of visual pursuit is open-loop (typically defined as the first 100 ms of pursuit) and is driven solely by feedforward mechanisms. The latter phase of smooth pursuit is closed-loop (occurring after 100 ms of pursuit) and is defined by online refinements of eye velocity and position based on sensory feedback processes encoded in the striate cortices and projected to posterior parietal cortices and then to medioposterior cerebellar lobules VI–VII (Ritchie, 1976; Fuchs et al., 1985; Stein, 1986; Kawato et al., 1987; Noda et al., 1990; Takagi et al., 1998; Chen-Harris et al., 2008).

Reduced pursuit accuracy has been documented in ASD during both the open-loop (Takarae et al., 2004a) and closed-loop phases (Takarae et al., 2004a; Aitkin et al., 2013) implicating both forward control and visual feedback processes. Deficits in open-loop pursuit were lateralized in ASD affecting only rightward movements. While suggesting a lateralized deficit, this finding (also present in unaffected parents) (Mosconi et al., 2010) is broadly consistent with diverse evidence indicating that hemispheric specialization for motor functions may be disrupted in patients (Escalante-Mead et al., 2003; Lindell and Hudry, 2013; Seery et al., 2013; Forrester et al., 2014). Further, the amplitude of saccades made to “catch up” to moving targets during pursuit also appears to be increased in ASD (Takarae et al., 2004a; Aitkin et al., 2013). However, it should be noted that some studies have found no differences in pursuit accuracy in ASD (Scharre and Creedon, 1992; Kemner et al., 2004), which may be related to findings that older individuals with ASD demonstrate more similar closed-loop tracking accuracy compared to healthy controls (Takarae et al., 2004a). Still, cortical-cerebellar dysfunctions appear to persist in adulthood as demonstrated by an fMRI study of smooth pursuit eye movements that found reduced activation in frontal eye fields, posterior partietal cortex, cingulate motor area, pre-supplemental motor cortex, and cerebellar lobules VI–VII in individuals with ASD (Takarae et al., 2007).

Perhaps the most sensitive probe of cerebellar circuits supporting oculomotor control is to systematically induce error into the system and then quantify the extent and rate at which the system adapts in order to evaluate plasticity in forward control mechanisms. Tests of saccadic adaptation have been used to assess cerebellar motor learning in non-human primates and patients with cerebellar lesions. In prototypical saccade adaptation tests, the visual target used to elicit a saccade is displaced by a consistent amplitude close to the time of movement initiation. Due to saccadic suppression of visual information during the movement, target displacement is seldom detected by the subject, but the amplitude of the saccade is adjusted over subsequent trials to land more closely to the displaced target rather than the original location. Adaptation mechanisms have been localized to the oculomotor vermis during this test (Desmurget et al., 1998; Barash et al., 1999), and reduced rates of adaptation and increased variability of saccade amplitudes have been found in individuals with cerebellar lesions that include the vermis (Golla et al., 2008; Xu-Wilson et al., 2009). Importantly, patients with non-vermal cerebellar damage show spared adaptation abilities.

Two recent ASD studies revealed reduced rates of saccade adaptation in affected individuals (Johnson et al., 2013; Mosconi et al., 2013). In addition, increased variability of saccade accuracy (Mosconi et al., 2013) and reduced time to peak saccade velocity (Johnson et al., 2013) also were reported. Importantly, Mosconi et al. (2013) found that 27% of subjects with ASD failed to show any level of adaptation compared to only 6% of controls, suggesting that a subset of patients may show more severe defects in cerebellar learning processes.

Studies of oculomotor control in ASD thus suggest alterations within cortical-ponto-cerebellar circuits involving the posterior vermis. These dysfunctions appear to be familial. Mosconi et al. (2010) demonstrated that unaffected family members of individuals with ASD show profiles of eye movement abnormalities similar to those described in individuals with ASD. Specifically, this study reported increased saccade error and saccade error variability and reduced pursuit accuracy during both closed- and open-loop phases suggesting that defects of cortical-cerebellar circuits involved in oculomotor control may contribute to the pathophysiology of ASD. Studies assessing the extent to which these deficits co-segregate within different families will be important for determining their utility as endophenotypes in family genetic studies. Similarly, studies showing direct linkages between these sensorimotor alterations and pathology in discrete cerebellar circuits may help sort out heterogeneity in the syndrome of autism based on biological parameters for which there is mechanistic understanding.

## Upper limb and manual motor control in ASD

Control of upper limb movement and force generation is supported by frontoparietal cortices and their targets in the cerebellar cortex and deep nuclei. The circuits that control various body parts are segregated at the levels of neocortex, cerebellar cortex, and within the deep nuclei (Grodd et al., 2001). Upper limb movements primarily involve anterior lobules I–V as well as more lateral areas of lobules V–VI extending into Crus I–II (Vaillancourt et al., 2006). Within these circuits, separate zones have been found to be differentially involved in controlling the amplitude, duration, and timing of movements (Mai et al., 1988; Spraker et al., 2012; Neely et al., 2013).

The most prominent feature of upper limb movements and manual motor control in patients with cerebellar lesions is dysmetria which frequently is characterized by overshooting of the target (Flament and Hore, 1986; Goodkin et al., 1993). Patients also show increased accuracy variability from trial to trial, impaired timing of their movements, overall slowness, and increased curvature of movement trajectories (Hallett et al., 1975; Bares et al., 2010). Upper limb and manual motor deficits are associated with atrophy of the intermediate and lateral cerebellum. Upper limb ataxia is found in patients with lesions of lobules IV–VI, whereas lower limb ataxia appears to result from defects in lobules III–IV (Schoch et al., 2006). Limb ataxia also is correlated with lesions affecting the interposed or dorsomedial dentate nuclei.

Limb movement abnormalities consistent with those seen in cerebellar patients have been found in individuals with ASD. When reaching toward targets, arm movements of individuals with ASD show increased temporal and spatial variability as well as atypical kinematic profiles characterized by reduced velocities and rates of acceleration as well as increased latencies of peak velocity (Glazebrook et al., 2006, 2009). The authors hypothesized that individuals with ASD compensate for deficits in forward control by slowing their reach and allowing more time for sensory feedback control processes to help guide the movement. Similar deficits in forward control have been shown for children with ASD when reaching for objects on large (easy) or small (hard) targets (Fabbri-Destro et al., 2009). While control subjects slowed the speed of their movement when reaching for smaller objects (in the more difficult condition), individuals with ASD failed to modulate their movement speed to account for the increased difficulty of the task. This finding suggests a compromised ability to appropriately modulate the action plan according to different task conditions.

Deficits in the sustained control of reaching movements also have been found in ASD. Glazebrook et al. (2009) found that reaching movements of individuals with ASD were more severely affected when they required greater visual-proprioceptive integration. These results suggest that individuals with ASD show a reduced ability to simultaneously process and integrate multisensory information, a process involving posterior cerebellar circuits that translate sensory feedback information into refined motor commands (Stein and Glickstein, 1992). Further, Gowen and Miall (2005) showed that individuals with ASD do not benefit from increased movement time in terms of their end-point accuracy during rapid targeted pointing. A more recent analysis of sinusoidal arm movements in ASD similarly found that patients show atypical kinematic profiles characterized by decreased movement smoothness (Cook et al., 2013). Unlike targeted movements, these oscillatory movements of individuals with ASD were increased in velocity and rate of acceleration. One possible explanation for the reduced smoothness of patients' movements is a failure to anticipate the point at which they must change the direction of their movement, or difficulties using predictive mechanisms to modulate action kinematics. Further, it is possible that patients were not able to precisely and consistently modulate the timing of the onset and offset of agonist and antagonist muscles across joints to facilitate smoother movements (Vilis and Hore, 1980; Nowak et al., 2004).

### Prehension

Prehension involves the coordinated act of reaching and grasping, and it is central to many daily living activities that are difficult for individuals with ASD (Brisson et al., 2012; Mulligan and White, 2012; Libertus et al., 2014). The ballistic acts of reaching and positioning the hand, affected by upper arm and forearm musculature, are largely independent from mechanisms subserving grasping actions, i.e., hand opening and then closing upon the object (Jeannerod, 1981, 1984). The two neural channels are assumed to be activated in parallel so that they can be functionally coupled during the act of reaching to grasp (Jeannerod, 1981, 1984). The “arm reaching” channel is believed to extract information about the spatial location of the object for transformation into motor patterns that bring the hand appropriately toward the object. The “grasp” channel extracts information about the intrinsic properties of the object for the determination of a suitable grasping position and appropriate level of force generation.

Slower reach-to-grasp movements have been found in individuals with ASD and comorbid intellectual disability (Mari et al., 2003). These movements were characterized by longer durations, greater temporal delay between peak reach velocity and peak grip aperture, a prolonged deceleration phase, reduced reaching velocity and prolonged time to maximum hand aperture. However, these deficits in forward control and movement coordination were not evident in individuals with ASD whose IQ was in the average range, suggesting that cerebellar dysfunctions may vary across the autism spectrum in relation to general cognitive ability. Still, others have found that individuals with ASD decompose reaching and grasping movements, and that these deficits are evident across the autism and IQ spectra (Cattaneo et al., 2007; Fabbri-Destro et al., 2009). These findings suggest that instead of translating movement goals into a chain of smoothly synchronized motor acts, individuals with ASD independently execute each component of the goal in a more sequential manner (Mari et al., 2003; Nazarali et al., 2009).

Studies of the grasping component of prehension provide a unique opportunity to investigate distinct motor control mechanisms in ASD that are linked to different circuits of the cerebellum. Neely et al. (2013) identified separate cerebellar circuits associated with dynamic aspects of gripping and sustained feedback control of grip. Spraker et al. (2012) also found that cerebellar regions that scaled with force amplitude could be segregated from those associated with the duration of force output. By examining different components of precision grip control in ASD, it thus may be possible to localize cerebellar circuit dysfunctions.

Prior studies of patients with cerebellar lesions have documented patterns of deficit during precision gripping including excess initial force output, increased sustained force variability, and decreased rates of force relaxation (Mai et al., 1988; Müller and Dichgans, 1994; Serrien and Wiesendanger, 1999; Fellows et al., 2001; Nowak et al., 2002, 2004). Forward control deficits would limit individuals' ability to form accurate initial movements, and sensory feedback processes would need to compensate to steer the movement back to the intended goal following afferent delays. Given the delays in sensory feedback, movement corrections can never be optimal (increased movement variability), because they are always computed for a portion of the trajectory that occurred in the past. Thus, a strategy emphasizing feedback over forward control processes may contribute to reduced precision or increased variability of motor output (Bastian, 2006).

Precision gripping studies have been performed to analyze forward control and feedback mechanisms. When an object is lifted using a pinch grip, grip force (against the object) increases simultaneously with load force (lifting of the object) prior to picking up the object (Johansson and Westling, 1984, 1988; Westling and Johansson, 1984). The rate of grip force increase and the grip force amplitude each depend on the object's weight and its surface texture. Therefore, grip force must be timed correctly with respect to the *anticipated* object load. During a test of precision gripping, Johansson and Westling (1984) found that participants' final grip force was greater when lifting heavier or more slippery objects suggesting that increases in grip force during the loading phase are planned in anticipation of the properties of the object.

Assessing grip and load force timing in individuals with ASD, David et al. (2009, 2012) found significant increases in grip to load force onset intervals suggesting temporal dyscoordination of these component processes. The authors also documented increased grip force at load force onset that may reflect either patients' inability to correctly time the grip force with respect to the anticipated load, or a compromised ability to use prior experience to correctly predict the required load force. In contrast, Gowen and Miall (2005) failed to identify forward control deficits in adults with ASD during precision gripping. Differences between these findings could reflect the non-overlapping age distributions across the studies. Gowen and Miall studied an older cohort (ages 18–49 years), and recent work has suggested that deficits in forward control of precision gripping may become less pronounced with age in ASD (Mosconi et al., 2015). Further, the nature of the different tasks used in these studies could have led to the discrepant findings. Gowen and Miall (2005) had subjects lift an object repeatedly allowing them to have greater experience with the load force required and thus providing sufficient information for individuals with ASD to adjust their force output. This would suggest that patients may be able to accurately calibrate forward controllers, but that they may need more practice than healthy individuals.

Using a novel analysis approach adopted from prior reaching studies, Wang et al. (2015) recently found that individuals with ASD show distinct patterns of initial gripping strategies. By measuring the derivatives of individual force traces, the authors identified inflections in force output reflecting changes to the initial motor plan putatively based on visual, somatosensory, and proprioceptive feedback inputs (Novak et al., 2000; Wisleder and Dounskaia, 2007; Grafton and Tunik, 2011). Three different types of feedforward control processes were identified: Type 1 pulses were associated with target overshooting and were characterized by rapid increases in force followed by a rapid force reduction to match the target; Type 2 pulses were defined by a more gradual increase in force followed by a pause and then a secondary force increase to reach the target; Type 3 pulses were distinguished by a series of temporally overlapping pulses used to reach the target force level. While controls showed a bias toward Type 1 pulses at low target force levels and trials that were shorter in duration, they utilized the more efficient Type 2 pulses to gradually reach their target levels when the target force or trial duration were increased. Individuals with ASD, in contrast, did not adapt their primary force strategy as flexibly, showing a persistent bias toward Type 1 pulses at both higher force levels and during trials of longer duration. These findings suggest that the internal representations used to predictively guide initial motor output are more stereotyped for individuals with ASD and thus may limit their ability to adapt motor skills to new and more complex task demands.

Further evidence for deficits in anticipatory control of motor output in ASD is seen in an analysis of force relaxation during precision gripping (Wang et al., 2015). The authors found that individuals with ASD show reduced rates of force relaxation after receiving visual cues that they should release their force level. To initiate grip relaxation, antagonist, and agonist muscles of the fingers must be synchronously activated and deactivated, respectively, within a rapid time frame. Reduced rates of grip force relaxation in ASD may reflect impairments in generating or executing coordinated muscle activities as seen in patients with cerebellar lesions (Küper et al., 2011). Studies directly examining muscle activities during precision gripping using electromyography (EMG) will be important for elucidating the mechanisms contributing to deficits in anticipatory motor processes in ASD.

**Figure 3:**
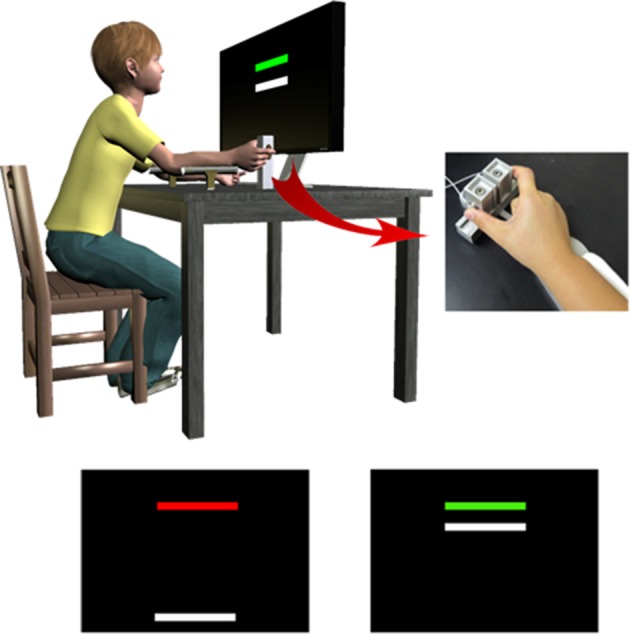
**To assess precision gripping control during rise, sustained, and relaxation phases, individuals pressed against two opposing load cells while receiving visual feedback from the monitor in front of them**. Individuals viewed two horizontal bars: a red/green target bar and a white force bar. The white force bar moved upward with increased force, and individuals were instructed to press on the load cells as quickly as possible when the target bar turned green so that the force bar reached the height of the target bar. They also were instructed to keep the force bar as close as possible to the target bar until the target bar turned red again, and then to release the load cells as fast as possible. Adapted with permission from Wang et al. (2015).

Feedback control of sustained precision grip also appears to be disrupted in ASD. During sustained control of grip, visual feedback information from visual cortex is projected to posterior parietal cortex, and then anterior to premotor and primary cortices (Glickstein and Stein, 1991). A more efficient subcortical route through posterior cerebellum also is used to translate visual-spatial feedback information into a more precise motor command executed by motor cortex (Glickstein and Stein, 1991; Stein and Glickstein, 1992). Mosconi et al. (2015) and Wang et al. (2015) each found that individuals with ASD show increased sustained force variability during a precision gripping test in which they receive online visual feedback about their performance (Figure 3). In these studies, participants pressed on two opposing load cells with their thumb and index finger while a horizontal force bar moved upwards with increased force. They were instructed to press on the load cells so that the force bar reached the same level as a static target bar, and then to keep it there as steadily as possible. Individuals with ASD showed increased variability of their force output over time that became more severe at higher force levels and in relation to the gain of visual feedback (Mosconi et al., 2015). Elevations in force variability were evident both at the lowest and highest visual gains, suggesting that individuals with ASD have a reduced ability to process highly degraded and highly magnified visual feedback information.

Mosconi et al. (2015) also found that individuals with ASD showed less complexity in the time-dependent structure of their force output, suggesting a failure to utilize the multiple control processes required to rapidly and precisely adjust motor behavior, including visual, proprioceptive, somatosensory, and forward mechanisms. Analyses of spectral profiles identified increased power in the 0–4 Hz range for individuals with ASD and relatively decreased power at higher frequencies (4–12 Hz) indicating an overreliance on slower feedback mechanisms. This is an inefficient strategy during large force productions for which rapid corrections are necessary to reduce larger errors in behavioral outputs. As the time delay of the motor response is increased, there would be a greater grip force deviation from the target if slower mechanisms are used exclusively. Therefore, individuals with ASD appear to show central deficits in integrating sensory feedback information and dynamically adjusting motor output consistent with defects of neocortical-posterior cerebellar circuitry. Thus, deficits in feedback control processes supporting online motor adjustments also appear to be present in ASD.

### Motor learning

Cerebellar processes involved in learning and updating internal action representations of upper limb movements appear to be compromised in ASD. During adaptation, error signals relayed via climbing fiber inputs to Purkinje cells invoke LTD that modifies the strength of population firing of GABAergic output from zones of Purkinje cells (Antunes and De Schutter, 2012). Initial studies of adaptation suggested that cerebellar learning occurs at a similar rate in ASD relative to healthy controls (Mostofsky et al., 2004; Gidley Larson et al., 2008). However, subsequent studies have indicated that individuals with ASD acquire new motor skills differently than controls. In a series of studies, Mostofsky and colleagues had participants complete a reaching adaptation task in which they moved a robotic handle toward a target. During this test, participants' moving arm was shielded from view, but they received visual feedback on a screen in front of them about the location of the handle and the target. Force perturbations were introduced perpendicular to the moving arm, and subjects thus changed the trajectory of their movement to counteract these forces and move as directly as possible to the target on subsequent trials. Participants then completed trials without force perturbations in which they demonstrated their ability to generalize their learned movement trajectories using an identical joint rotation (proprioceptive feedback) or hand motion (visual feedback) as the training movements. Haswell et al. (2009) and Izawa et al. (2012b) each found that individuals with ASD generalized their movements in proprioceptive coordinates to a greater extent than controls suggesting an overreliance on proprioceptive feedback information. Izawa et al. (2012b) and Marko et al. (2015) also found that individuals with ASD showed weaker generalization in visual space compared to controls implicating a reduced ability to integrate visual feedback information during motor learning. The latter study showed that reduced learning rates in ASD in visual coordinates were related to reduced volumes of anterior cerebellar lobules extending into lobules VI and VIII. Further, patients' over-reliance on proprioceptive feedback in acquiring new motor skills was associated with social and imitation impairments suggesting that fundamental deficits in motor control and learning may contribute to deficits in more complex social-motor skills in ASD (Haswell et al., 2009; Cook et al., 2013).

In summary, studies of upper limb movements, manual motor control, and motor learning implicate defects of forward control, sensory feedback control, and cerebellar dependent learning in ASD. These processes are supported by distinct zones of the cerebellum and their interactions with frontal and parietal cortices. Few functional MRI studies have examined cortical-cerebellar contributions to upper limb control. Allen and Courchesne (2003), Allen et al. (2004) each found increased anterior cerebellar activation and atypical contralateral and posterior cerebellar activation in subjects with ASD during a self-paced finger tapping test. During finger tapping tests in which participants follow a visual prompt, anterior, and ipsilateral cerebellar lobules show reduced activation in ASD (Müller et al., 2001; Mostofsky et al., 2009). Further, reduced activation was seen in thalamic and motor cortical targets in ASD and reduced functional connectivity between these motor regions also was documented (Müller et al., 2001; Mostofsky et al., 2009). Anterior cerebellar lobules IV–VI and their connections with frontal and parietal motor regions thus appear to be compromised in ASD. These effects may disrupt control and learning of upper limb and manual motor actions, and they could impact the development of more complex social motor skills that are central to the disorder.

## Gait and postural control in ASD

Cerebellar circuits involved in controlling balance and gait have been identified in the vermis and intermediate cerebellum (Haines and Mihailoff, 2002; Apps and Garwicz, 2005; Ramnani, 2006). These regions receive afferent input both from motor and parietal cortices as well as direct innervation from the spinal cord (Apps and Garwicz, 2005). Afferent relays to the spinocerebellum originate from interneurons in the spinal gray matter that terminate as mossy fiber inputs in the vermis or intermediate cortex (Apps and Garwicz, 2005; Ramnani, 2006). Spinocerebellar inputs provide rapid proprioceptive feedback information that can be integrated with somatosensory, visual and vestibular feedback to maintain postural stability. Based on these inputs, individuals are able to align the projection of their body's center of mass within their base of support by actively manipulating the center of pressure under their feet while standing (Riccio, 1993; Winter, 1995; Horak, 2006). During walking, ventral spinocerebellar tracks carry internally generated information about movement rate and trajectories as well as the rhythmic discharge of somatic receptors to the cerebellum whereas the dorsal track provides sensory feedback information during the movements (Jahn et al., 2004; Hoellinger et al., 2013).

During walking, patients with spinocerebellar atrophy show an increased postural sway path along the anterior-posterior axis (Diener et al., 1985). Increased stride width and stride length variability are hallmark signs of ataxia evident in the majority of cerebellar patients during walking (Cavallari et al., 2013; Kafri et al., 2013). Patients with anterior cerebellar lesions also demonstrate issues when trying to maintain postural control while standing. In response to externally triggered perturbations, patients with alcohol-induced anterior cerebellar lobe syndrome showed increases in the magnitude of their EMG responses and overshooting of their initial postural compensation suggesting imprecision of forward control mechanisms (Horak and Diener, 1994). Abnormal EMG latencies recorded from anterior tibial and triceps surae muscles also have been observed in patients with anterior lobe atrophy as well as those with vestibulo-cerebellar lesions and Friedreich's ataxia (primarily affecting the spinocerebellar pathway) in response to unexpected rotations of a platform on which they were standing (Diener et al., 1984). These results suggest alterations in sensory feedback control processes that reactively adjust muscular forces used to maintain postural stability. These deficits differ somewhat from those reported in patients with basal ganglia or motor cortical dysfunctions suggesting that the cerebellum plays a highly specialized role in forward and feedback control of postural and balance mechanisms, and in coordinating the timing and amplitude of movements during walking.

General balance preservation when standing is a continuous process driven primarily by lower limb muscular reflexes (a feedback control process) that involve minimal effort or attention (Woollacott and Shumway-Cook, 2002). When self-initiated (e.g., leaning forward) or externally triggered perturbations to the system are predicted, forward control processes are invoked to make anticipatory postural adjustments (APAs) and dynamically ensure balance (for details, see reviews by Massion, 1992; Aruin, 2002). For example, APAs allow an individual to release an object without falling over by initiating a compensatory backward sway through deactivation of postural flexor muscles (i.e., erector spinae and soleus) and activation of extensor muscles (i.e., rectus abdominis, rectus femoris, tibialis anterior), respectively (Aruin and Latash, 1995). Comparatively, if the load is slowly taken away by the experimenter, the backward postural sway and muscle activities will be attenuated or absent.

Few studies have examined APA mechanisms in ASD. Studying a small sample of children with ASD, Schmitz et al. (2003) found a unique pattern of atypical muscle activation/deactivation during a task of postural control. During this test, participants were seated in a chair with their left elbow flexed at 90° while their forearm rested on a support attached to the chair. A load was attached to a bracelet on the participant's left wrist. In trials of self-initiated unloading, participants unloaded the bracelet using their right hand when they felt ready. To stabilize their left forearm after unloading, healthy controls showed anticipatory adjustments involving activity in the biceps brachii 15 ms *before* unloading the weight. This change was followed by a stretch reflex of triceps brachii after the onset of unloading. In contrast, children with ASD showed delayed biceps brachii activation with its onset 58 ms *after* unloading and triceps activity attenuation throughout the trial. Results thus indicate that individuals with ASD are less able to use APA mechanisms to maintain postural control during self-initiated activities.

The neurophysiological substrates of APA have been examined in ASD using electroencephalogram (EEG) during imposed and self-initiated unloading tasks (Martineau et al., 2004). While EEG provides high frequency information on neocortical activity, it is less suitable for directly studying cerebellar mechanisms. However, given the known effects of cerebellar activity on motor and parietal cortical activity, analysis of EEG data during tests of APA and postural control may offer some insight into cortical-cerebellar functions in ASD (Manto et al., 2012).

During an externally-imposed unloading task, decreased power density at 6–8 Hz was observed after unloading corresponding to the cortical responses recorded during involuntary arm reflexes. This cortical response was identified bilaterally over motor cortices (i.e., C3 and C4) in both control children and children with ASD suggesting that unloading reflexes are intact in ASD (Schmitz et al., 2003). During a self-initiated bimanual unloading task, control children showed a significant decrease in power density above C3 and C4 400–500 ms before the onset of the action. However, children with ASD did not show corresponding anticipatory decreases in power density suggesting deficits in cortical systems involved in forward control mechanisms supporting APAs as well as possibly their cerebellar targets. However, this pattern of deficit appears to be different from what has been reported for patients with cerebellar lesions (Horak and Diener, 1994). While individuals with ASD show antagonist muscle activations that are largely delayed and depressed, postural corrections in patients with cerebellar lesions show a pattern of overshooting. Determining the mechanisms contributing to deficits in forward control processes involved in postural stability will be important for identifying abnormal circuits in ASD. This information may be particularly important in the context of findings that postural stability provides a critical foundation for a range of more complex and fine-grained motor behaviors.

During studies of balance, individuals with ASD have been found to show increased postural sway across the lifespan (Molloy et al., 2003; Minshew et al., 2004; Chang et al., 2010; Fournier et al., 2010a; Radonovich et al., 2013; Memari et al., 2014). An important observation from these studies is that children with ASD showed greater medio-lateral as opposed to anterior-posterior postural sway, and that their medio-lateral range of motion was greater than that for typically developing controls (Kohen-Raz et al., 1992; Chang et al., 2010; Fournier et al., 2010a; Memari et al., 2014). Increased postural sway in medio-lateral directions during quiet stance is commonly observed in young children under the age of 10 years who show a reduced ability to maintain balance in the anterior-posterior direction causing them to increase their base of support in medio-lateral directions to compensate from internal perturbations (Maki et al., 1990; Riach and Starkes, 1994; Slobounov and Newell, 1994; Prieto, 1996). Studies documenting postural instability in ASD thus suggest that this system remains immature throughout development (Minshew et al., 2004).

When required to stand as still as possible under naturalistic conditions, healthy controls primarily rely on somatosensory information, followed by vestibular and then visual feedback (Peterka, 2002; Horak, 2006). Increased weighting toward vestibular and visual information can be induced by reducing the stability of the standing surface and thus limiting somatosensory feedback (Massion, 1994; Peterka, 2002; Horak, 2006). When asked to stand on a foam board to provide an unstable surface, individuals with ASD showed increased variability of their center of pressure over time and a reduction of postural stability compared to healthy controls (Molloy et al., 2003). This deficit became more severe when participants kept their eyes shut suggesting a greater reliance on visual feedback control processes and a reduced ability to utilize vestibular information to help preserve balance.

During tests of dynamic standing in which participants attempt to maintain balance in a virtual environment with oscillatory visual feedback information, healthy controls are able to optimize the frequency of their postural sway to match that of visual feedback (Slobounov et al., 1997, 1998). In contrast, individuals with ASD showed attenuated postural sway at 0.2 Hz (Gepner et al., 1995; Gepner and Mestre, 2002). Greffou et al. (2012) also found that younger participants with ASD (ages 12–15 years old as opposed to 16–33 years old) displayed significantly less postural sway than controls when visual stimuli oscillated at 0.5 Hz. These findings indicate that visual feedback mechanisms used to help dynamically support postural control are compromised in individuals with ASD.

### Gait

At the initiation of walking, there is a purposeful uncoupling of the center of pressure and center of mass (Winter, 1983, 1995; Remelius et al., 2014). The center of pressure shifts posteriorly to generate forward momentum and propel the center of mass forward. The center of pressure also shifts initially toward the swing leg as a result of the unloading of the stance leg generating the initial lateral acceleration of the center of mass toward the stance leg (Winter, 1995). Studying gait initiation, (Fournier et al., 2010b) found that individuals with ASD showed intact center of pressure posterior shifts but a significantly reduced center of pressure lateral range of motion indicating a reduced capacity to generate lateral momentum and propel the upper body from side-to-side.

Walking has been described as a “throw-catch up” process during which the body propels the center of mass into an unstable state beyond the anterior limit of the base of support in what is often called a “controlled fall” (Winter, 1983, 1995; Remelius et al., 2014). The catch up process then involves taking a step forward to slow momentum and create a new base of support. Early studies of gait in ASD identified Parkinsonian features including dystonia, involuntary dyskinesia of the extremities, abnormalities in muscle tone, rigidity, hypertonia, and decreased coordination of arm and leg movements. Studying individuals with ASD, Vilensky et al. (1981) observed reduced stride length, increased stance duration, increased hip joint flexion at “toe-off” and reduced knee extension and ankle dorsiflexion at initial contact. Additional features reported in a series of observational studies included toe-striking, strides in which the whole foot was simultaneously placed on the ground at the phase of initial contact rather than as a heel-strike, “striatal” toes (i.e., spontaneous upward movement of the big toes similar to the Babinski reflex), claw toe and hand posture (Walker and Coleman, 1976; Folstein and Rutter, 1977; Damasio and Maurer, 1978; Teitelbaum et al., 1998; Esposito and Venuti, 2008). Vernazza-Martin et al. (2005) reported significant increases in ASD patients' head, shoulder, trunk, and hip angular motion that were associated with an increased variability in the trajectories of their walking paths. Increased stride length and stride width variability also have been commonly observed in individuals with ASD (Blin et al., 1990; Vernazza-Martin et al., 2005; Rinehart et al., 2006).

The mechanisms that contribute to walking abnormalities remain unclear as patterns do not cleanly fit with models of ataxia, basal ganglia dysfunction, or other neurological disorders. However, sensory processing and integration disturbances appear to play a significant role in the walking disturbances observed in individuals with ASD. Rinehart et al. (2006) studied participants with ASD who were instructed to walk either at their own preferred speed (stride length) or with strides that were 20% greater than their average stride length. For the longer stride condition, participants walked either utilizing visual cues indicating how long to stride, or without visual cues. In contrast to healthy controls, individuals with ASD showed significant increases in stride-length variability for both preferred and visually cued conditions indicating an impaired ability to consistently anticipate the amplitude of the targeted movement or to use visual feedback to guide the amplitude of the movement.

In summary, studies of posture, gait initiation and walking in ASD implicate gross motor impairments consistent with deficits of both forward control and sensory feedback mechanisms involving medial and intermediate cerebellar circuits. These deficits may reflect alterations in spinocerebellar tracts that innervate the cerebellum primarily through the inferior cerebellar peduncle (Cheng et al., 2010). Further studies examining spinocerebellar circuit anatomy and function are needed to better understand the integrity of these networks in ASD and their relation to gross motor abnormalities in patients.

## Early dysmaturation of sensorimotor systems in ASD

Multiple cerebellar circuits involved in sensorimotor control thus appear to be compromised in ASD. These circuits undergo rapid refinement during the early postnatal period that, if derailed, could have a significant impact both on sensorimotor behavior and other aspects of development (Ashwell and Zhang, 1992). The cerebellum undergoes rapid growth during the last trimester and early postnatal period. Mossy fibers form transient contacts with developing Purkinje cells during embryonic development, but they do not form their parasagittal zones with Purkinje cells until shortly after birth (Arsénio Nunes and Sotelo, 1985). Climbing fibers become organized into parasaggital stripes by late embryogenesis in the rat and mouse (Sotelo et al., 1984; Chedotal and Sotelo, 1992; Paradies et al., 1996). At a more macroscopic level, the cerebellum has been shown to undergo the greatest amount of volumetric increase among all studied brain regions during the first 30 days of life (Holland et al., 2014). Perinatal disruptions of neurodevelopment could selectively affect these processes that are occurring rapidly around birth.

To add to the vulnerability of the cerebellum, Purkinje cells are a relatively large (50–80 μm) class of neurons whose synapses with olivary climbing fibers form some of the most energy demanding connections in the brain (Sugimori and Llinás, 1990; O'hearn and Molliver, 1997; Welsh et al., 2002). As a result of the high level of excitatory amino acid synaptic connections and the response of the Purkinje cell that is mediated by voltage-gated and receptor-gated calcium channels, the Purkinje cell has an exceptionally high metabolic demand. This high metabolic demand combined with constant input from the inferior olive and large amounts of calcium stores and influx makes the cell particularly vulnerable to excessive rises in intracellular calcium that are associated with excitotoxicity and cell death (Vajda, 2002).

These factors may help explain the repeated findings of Purkinje cell pathology in ASD. Studies of other cerebellar cells provide insight into possible timing and mechanisms. Purkinje cells synapse with basket and stellate interneurons to support their survival. In the lone study to count the number of basket and stellate cells in brain tissue from individuals with ASD, no difference in the number of these interneurons were found suggesting that Purkinje cells were generated, migrated to their proper location and then atrophied or died (Whitney et al., 2009). There also is no apparent loss of climbing fibers from the inferior olive (Whitney et al., 2008). Climbing fibers synapse with Purkinje cells shortly before birth but die off if there is Purkinje cell loss (Holmes and Stewart, 1908; Whitney et al., 2008). Findings that there is no loss of climbing fiber inputs suggest that Purkinje cell loss occurs prenatally, rather than as a regressive effect of later alterations in cortical feedback. Consistent with this hypothesis, Bauman and Kemper (1985) report an absence of gliosis. However, Bailey et al. (1998) reported gliosis in a subset of post-mortem tissue of individuals with ASD that could be associated with postnatal loss of Purkinje cells. Further research is needed to resolve this important discrepancy.

Sensorimotor abilities also undergo rapid maturation during the early postnatal period. These skills form the basis for multiple aspects of cognitive and language development, and their disruption could directly contribute to the social-communication deficits that define ASD (Lebarton and Iverson, 2013). Sensorimotor impairments have been repeatedly shown to be associated with social and language impairments in ASD (Takarae et al., 2004b; Haswell et al., 2009; Mosconi et al., 2009, 2015; Cook et al., 2013) and variable in terms of their severity over the course of development (Takarae et al., 2004b; Luna et al., 2007; Mosconi et al., 2015). Both retrospective videotape analyses and prospective studies of infant siblings of children with ASD have documented abnormal sensorimotor development within the first year of life affecting postural control, crawling and early walking, fine motor movements, prehension, and eye movements (Brian et al., 2008; Lebarton and Iverson, 2013; Ben-Sasson and Gill, 2014; Leonard et al., 2014; Ozonoff et al., 2014; Sacrey et al., 2015). Evidence that sensorimotor deficits may be present before the defining features of the disorder further indicates that their study in infancy may be highly informative for early diagnostic efforts aimed at guiding early interventions.

## Preclinical modeling of cerebellar involvement in ASD

In addition to being affected in idiopathic ASD, the cerebellum has been consistently implicated in several monogenetic syndromes associated with ASD (e.g., FXS, Phelan-McDermid Syndrome). And, its disruption appears to be selectively related to the severity of patients' ASD symptoms (Kaufmann et al., 2003; Eluvathingal et al., 2006; Aldinger et al., 2013). These findings suggest that preclinical genetic models may advance a more mechanistic understanding of the cerebellum's contributions to the pathogenesis of ASD. Preclinical models also provide a means to develop and ultimately test targeted therapeutics that will benefit sensorimotor and behavioral dysfunctions in ASD. To this end, multiple preclinical models have emerged that have shed light on the underlying pathophysiology of ASD. Here, we will focus on several models of known monogenetic disorders in which a disproportionate number of patients meet criteria for ASD, and chromosomal or gene abnormalities associated with high rates of ASD. Sensorimotor impairments have been reported for many of these models, and cognitive and behavioral dysfunctions consistent with and pathognomonic for cerebellar dysfunction also have been reported.

### Fragile X syndrome (FXS)

FXS is caused by expansion of CGG trinucleotide repeats in the Fragile X mental retardation 1 (FMR1) gene, which codes for the Fragile X Mental Retardation Protein 1 (FMRP1). Resulting methylation of the FMR1 promoter results in the absence of functional FMRP1 protein product. Individuals with FXS show developmental/intellectual disability and high rates of ASD and account for ~1–2% of total ASD cases. Fmr1 knockout mice display hyperactivity, repetitive behaviors, impaired learning and memory, and variable social impairments (1994; Koekkoek et al., 2005; Spencer et al., 2005; McNaughton et al., 2008; Moy et al., 2009). Mutant mice also demonstrate cerebellar abnormalities with elongated spines and enhanced LTD (Koekkoek et al., 2005) consistent with enhanced plasticity in other brain regions (Bear et al., 2004). Fmr1 global knockout mice as well as Purkinje cell specific fmr1 knockouts demonstrate impairments in eyeblink conditioning, a form of associative learning requiring intact cerebellar function (Freeman and Steinmetz, 2011). Patients with FXS demonstrate similar deficits in eyeblink conditioning (Koekkoek et al., 2005) as well as cerebellar-associated motor dysfunctions (Zingerevich et al., 2009). Taken together with studies showing abnormal eyeblink conditioning in human studies of patients with idiopathic ASD (Sears et al., 1994; Oristaglio et al., 2013), these data support the presence of abnormal cerebellar function in patients with idiopathic ASD and ASD associated with Fragile X disorders.

### Tuberous sclerosis complex (TSC)

TSC, like FXS, is a monogenetic disorder associated with intellectual and neurodevelopmental disability, seizures, and ASD (~50%), contributing to 1–2% of ASD patients. This disorder results from mutations of a single copy of either TSC1 or TSC2, whose protein products heterodimerize and act to negatively regulate the mechanistic target of rapamycin (mTOR) protein, a critical regulator of protein translation (Kelleher and Bear, 2008; Thoreen et al., 2012). Patients with TSC show fine motor impairments (Jeste and Geschwind, 2014), and patients with mutations in the TSC2 gene have demonstrably smaller cerebella (Weisenfeld et al., 2013). Moreover, cerebellar lesions associated with ASD in TSC and the deep cerebellar nuclei have been found to be abnormal in patients with ASD and TSC (Weber et al., 2000; Eluvathingal et al., 2006), suggesting that cerebellar dysfunction may play a selective role in the pathogenesis of ASD in TSC (Asano et al., 2001).

To better understand whether cerebellar dysfunction was sufficient to cause ASD behaviors, Tsai et al generated a mouse model lacking tsc1 in cerebellar Purkinje neurons. These mutant mice demonstrated behaviors associated with ASD—social impairment, repetitive behavior, inflexible behavior, vocalization abnormalities—in addition to electrophysiologic abnormalities and cellular pathology similar to that seen in TSC patients (Tsai et al., 2012). In addition, abnormalities in delayed eyeblink conditioning similar to those seen in patients with ASD and FXS are seen in these mice (Kloth et al., 2015). Early postnatal treatment of these mutant mice with the mTOR specific inhibitor rapamycin prevented the development of motor and ASD behaviors and the development of cerebellar pathology (Tsai et al., 2012). These findings were subsequently replicated in a mouse model where tsc2 was deleted in cerebellar Purkinje cells (Reith et al., 2013). Whether later treatment will be efficacious in treatment of these abnormalities remains an interesting avenue for further study.

### Shank 3

SH3 and multiple ankyrin repeats 3 (Shank3)/proline rich synapse associated protein 2 (ProSap2) has been implicated to be the critical, pathologic gene responsible for Phelan-McDermid Syndrome in patients with 22q13 deletions/mutations. Phelan-McDermid Syndrome is characterized by elevated rates of neurodevelopmental disability, seizures, sleep disorders, and ASD (Phelan and McDermid, 2012; Soorya et al., 2013; Sarasua et al., 2014). Although mutations can be causal in Phelan-McDermid Syndrome, SHANK3 mutations have also been identified in several cases of idiopathic ASD. SHANK3 is a postsynaptic scaffolding protein that plays critical roles in excitatory synaptic transmission (Zoghbi and Bear, 2012) and is expressed widely throughout the brain, including the cerebellum (Peça et al., 2011). Rodent models with loss of Shank3 display social impairments, repetitive behaviors, abnormal vocalizations, and impaired learning (Bozdagi et al., 2010; Peça et al., 2011; Wang et al., 2011). Although the precise role for cerebellar Shank3 has not been studied, Shank3 mutant mice demonstrate significant abnormalities in cerebellar anatomy (Ellegood et al., 2014) and deficits in cerebellar function with impaired delayed eyeblink conditioning (Wang et al., 2014).

### 15q11-13 duplication

15q11-13 duplication has been identified in up to 3% of cases of ASD, making it the most frequently identified chromosomal abnormality in ASD (Urraca et al., 2013). This region is genetically complex as maternal deletion of the region results in Angelman's Syndrome while paternal deletion results in Prader-Willi syndrome. Patients with 15q11-13 duplications have elevated rates of motor dysfunction (Urraca et al., 2013), while both deletion related syndromes (Angelman's and Prader-Willi) also are characterized by profound motor abnormalities (Buiting, 2010). Mouse models of paternal 15q11-13 duplication demonstrate autistic-like behaviors including social dysfunction, behavioral rigidity, and abnormal vocalizations (Nakatani et al., 2009). To investigate cerebellar contributions to these behaviors, Piochon et al. (2014) examined these mice and identified abnormalities in motor learning, reduced eye blink conditioning, impaired parallel fiber-Purkinje cell LTD, and impaired elimination of surplus climbing fiber inputs to Purkinje cells.

### Neuroligin 3

Cerebellar dysfunction also has been implicated in models of nonsyndromic ASD. Neuroligin 3 (nl3) encodes a postsynaptic adhesion molecule involved in synapse assembly (Südhof, 2008). Mutations (both point mutations and deletions) have been identified in ASD patients (Jamain et al., 2003; Levy et al., 2011; Sanders et al., 2011). Nl3 mutant mice (either point mutation knockin or deletions) demonstrate autistic-like behaviors including motor coordination impairments, social impairments, repetitive behaviors, and abnormal vocalizations (Baudouin et al., 2012), while mice with a nl3 knockin mutation demonstrate changes in cerebellar anatomy on MRI (Steadman et al., 2014). Purkinje cell specific nl3 mutant mice demonstrate increased hyperactivity (Rothwell et al., 2014), while Purkinje cell specific expression of nl3 rescued motor coordination deficits in knockout mice, consistent with a critical role for cerebellar nl3 in the pathogenesis of ASD behaviors (Baudouin et al., 2012).

### Engrailed 2

ENGRAILED 2 (En2) is a homeobox transcription factor that has been implicated in ASD through numerous genetic association studies (Gharani et al., 2004; Benayed et al., 2005). En2 is highly expressed in the cerebellum and abnormalities in En2 expression levels have been identified from postmortem ASD cerebellum (James et al., 2013; Choi et al., 2014). En2 transgenic mice demonstrate abnormal cerebellar development while En2 knockout mice display reduced cerebellar volumes, reduced Purkinje neuronal numbers, and abnormalities in cerebellar foliation (Millen et al., 1994; Ellegood et al., 2014). Knockout mice display motor and social impairments but demonstrate normal vocalizations and grooming behaviors (Brielmaier et al., 2012). They also demonstrate reductions in noradrenergic levels. When targeted with norepinephrine reuptake inhibitor therapy, amelioration of abnormal behaviors results, suggesting a potential avenue of targeted therapy (Brielmaier et al., 2014).

### CADM1

Cell Adhesion molecule 1 (Cadm1) is a synaptic cell adhesion molecule that has been identified as a rare genetic cause of ASD (Zhiling et al., 2008). Cadm1 is highly expressed in the dendritic arbor of Purkinje neurons and Cadm1 knockout mice demonstrate reductions in cerebellar size (Fujita et al., 2012) and abnormal social behaviors, abnormal vocalizations, increased anxiety, and abnormal motor coordination (Takayanagi et al., 2010; Fujita et al., 2012).

### RORα

Retinoic Acid receptor-related orphan receptor alpha (Rorα) has been implicated in sporadic cases of ASD. The naturally occurring staggerer mouse has a mutation in this gene, and as its name suggests, it displays profound motor dysfunction and ataxia (Sotelo and Changeux, 1974; Steinmayr et al., 1998). Loss of this gene results in subsequent cerebellar hypoplasia with marked loss of Purkinje neurons (~80%) and a comparable loss of granule cells. In addition to motor deficits, these mice demonstrate abnormal learning and aberrant responses to novel stimuli (Goldowitz and Koch, 1986; Lalonde et al., 1996a,b), although these abnormalities are potentially adversely affected by the profound motor dysfunction seen in these animals.

### Integrin 3

Hyperserotonemia has been identified in ~30% of sporadic ASD cases, and Integrin 3β (ITGB3) has been implicated in genetic regulation of serotonin levels through interactions with serotonin transporters. Certain haplotypes of itgb3 are associated with ASD (Weiss et al., 2006; Napolioni et al., 2011) while a mutation in itgb3 has been identified in a patient with ASD (O'roak et al., 2012). Because of critical roles for itgb3 in platelet function, autistic behaviors in knockout models have not been fully evaluated, although cerebellar anatomy has been shown to be reduced in these mutant mice (Steadman et al., 2014). However, heterozygous itgb3 mutants display both abnormal social behaviors and increased repetitive behaviors (Carter et al., 2011), implicating potential roles for cerebellar ITGB3 in the pathogenesis of ASD.

### Valproic acid

Environmental exposures have also been linked to elevated ASD risk. One such model that has been widely examined is exposure to valproic acid (VPA). VPA is widely used as an antiepileptic medication and/or for its mood stabilization properties in the treatment of bipolar disorders. *In utero* exposure to VPA during the first trimester has been linked to increased risk of congenital malformations including neural tube defects but has also been linked to increased risk of ASD development (reviewed in Roullet et al., 2013). Numerous rodent models of VPA exposure have demonstrated impairments in motor, social, and communication behaviors in addition to increased repetitive behaviors (Roullet et al., 2013). Reductions in Purkinje cell numbers and cerebellar size have been demonstrated in these models (Ingram et al., 2000), and abnormal eye blink conditioning has been observed in mice with early VPA exposure (Stanton et al., 2007). Interestingly, motor activity has been reported to ameliorate VPA induced behavioral alterations while exposure to the antioxidant piperine has been reported to ameliorate behavior and pathology in the cerebellum (Pragnya et al., 2014). As markers of oxidative stress are noted to be increased in the cerebellum in postmortem studies of ASD brains (Sajdel-Sulkowska et al., 2009, 2011) and in genetic models of ASD (Tsai et al., 2012), these findings suggest the possibility of shared mechanisms in the pathogenesis of ASD.

## Future studies

Findings from histopathology, gene expression, *in vivo* imaging and sensorimotor studies each suggest a critical role for the cerebellum in the pathophysiology of ASD. Comparisons across studies suggest that different cerebellar circuits may be variably affected in different patients. Given the crucial problem of resolving biological heterogeneity in ASD, clarification of patterns of altered function in these different circuits may provide a much needed window into biological mechanisms affecting different patients or patient subgroups and their clinical implications. Studies aimed at determining the regional specificity of cerebellar pathology across different lobules and subregions are necessary for identifying whether cerebellar defects are more diffuse or specific to distinct circuits. Comprehensive assessments of multiple distinct sensorimotor abilities and their development over early childhood in ASD are needed. Further, integrated analyses across different levels including combined preclinical and clinical assessments are needed to better understand how genetic and molecular processes relate to cellular and brain system anomalies as well as clinical symptoms.

Rodent models of ASD provide an important means through which the contribution of cerebellar dysfunction to the pathogenesis of ASDs can be better understood. However, most studies thus far have been limited to describing evidence of cerebellar dysfunction in global models of ASD (Zingerevich et al., 2009; Baudouin et al., 2012; Roullet et al., 2013; Brielmaier et al., 2014; Ellegood et al., 2014; Piochon et al., 2014; Steadman et al., 2014). Only a few studies have specifically examined the effects of targeted disruption of cerebellar neurons on sensorimotor functions, cerebellar learning paradigms such as eye blink conditioning (Koekkoek et al., 2005; Kloth et al., 2015), ASD defining behaviors (Tsai et al., 2012; Reith et al., 2013), and other behaviors associated with neurodevelopmental disorders (Rothwell et al., 2014). As these models are further evaluated, the contributory role for the cerebellum in motor and non-motor behavioral dysfunction can be elucidated.

The neuronal circuitry by which the cerebellum regulates these diverse behaviors and aspects of development also remains to be further clarified. Studies demonstrate connections between the cerebellum and medial prefrontal cortices in rodents and primates, with ASD models demonstrating dysfunction in these cerebellar mediated circuits (Rogers et al., 2011, 2013; Bostan et al., 2013). Technology has been developed to pair genetic tools with neuromodulatory paradigms through chemical means (Sternson and Roth, 2014) or light based approaches (Steinberg et al., 2015) for use *in vitro* and for *in vivo* preclinical animal studies. These technologies provide a promising approach for teasing apart circuit based mechanisms underlying complex behaviors, such as those dysregulated in ASD. In combination with cerebellar targeting, these technologies offer intriguing potential to identify the pathogenic cerebellar circuits mediating behaviors that are dysregulated in neurodevelopmental disorders such as ASD, and they raise the possibility of targeted, cerebellar mediated therapeutic development.

Mouse models, as demonstrated, provide a powerful model system to further explore the contribution of cerebellar dysfunction to sensorimotor and cognitive/behavioral dysfunction in patients with ASD. With sensorimotor behavioral paradigms in combination with cerebellar learning modalities (eye blink conditioning) and cerebellar mediated reflexes (oculocephalic reflex), these models provide tools to better delineate cerebellar dysfunction in ASD. Considering that some of these paradigms—motor function, eye blink conditioning as examples—can be tested during early development or even during the neonatal period (Little et al., 1984; Fifer et al., 2010), these modalities could also emerge as biomarkers that could contribute to early ASD diagnosis.

## Summary

Converging clinical and preclinical data identify an important role for the cerebellum in the pathogenesis of both monogenic syndromes associated with ASD and idiopathic ASD. These data identify anatomical and functional alterations of multiple distinct cerebellar circuits involving areas of neocortex and subcortical regions such as the basal ganglia. The cerebellum's rapid postnatal growth suggests that there may be critical periods of development during which it helps to scaffold the specialization of association cortices and other later developing brain systems (Rice and Barone, 2000). While gene expression studies show that ASD implicated networks are highly expressed in postnatal cerebellum, the timing of cerebellar defects across circuits has not yet been assessed. Narrowing the window during which these genetic and epigenetic events may disrupt cerebellar maturation will be critical for developing biomarkers and effective therapeutics for different forms of ASD. Evidence from preclinical model studies shows the possibility for selective rescue of cerebellar pathology in TSC (Tsai et al., 2012) and perhaps other forms of ASD. Increased attention to the role of cerebellar pathology in sensorimotor and other ASD-related behaviors may thus provide critical insights into pathogenic mechanisms as well as novel targeted molecular, cellular, and anatomic based therapeutics.

## Author contributions

All authors contributed to the conception of this work and revising it critically for important intellectual content. All authors provided final approval of the version to be published and agree to be accountable for all aspects of the work including ensuring that questions related to the accuracy or integrity of any part of the work be appropriately resolved. MM contributed to drafting, organizing, and critically evaluating all sections of this paper. ZW contributed to drafting sections relating to upper limb control and gait/posture. LS contributed to drafting the oculomotor section. PT contributed to drafting the section on preclinical modeling.

## Funding

This study was supported by NIMH 092696, NINDS 083733, NICHD Autism Center of Excellence award 055751, and Autism Speaks.

### Conflict of interest statement

John A. Sweeney served on advisory boards for Roche, Takeda, BMS, and Lilly. The Editor Mustafa Sahin declares that, despite collaborating on two articles with Peter Tsai in 2014, the review was conducted objectively and no conflict of interest exists. The other authors declare that the research was conducted in the absence of any commercial or financial relationships that could be construed as a potential conflict of interest.
